# The Impact of Social Structures on Deviant Behaviors: The Study of 402 High Risk Street Drug Users in Iran

**DOI:** 10.1155/2016/6891751

**Published:** 2016-11-29

**Authors:** Maryam Mehrabi, Sharareh Eskandarieh, Mahmoud Khodadost, Maneli Sadeghi, Ali Nikfarjam, Ahmad Hajebi

**Affiliations:** ^1^Department of Mental, Social Health and Addiction, Ministry of Health, Tehran, Iran; ^2^Health and Medical Sociology Group of Iranian Sociology Association, Tehran, Iran; ^3^Brain and Spinal Cord Injury Research Center, Neuroscience Institute, Tehran University of Medical Sciences, Tehran, Iran; ^4^MS Research Center, Neuroscience Institute, Tehran University of Medical Sciences, Tehran, Iran; ^5^Student Research Committee, Zabol University of Medical Sciences, Zabol, Iran; ^6^Department of Epidemiology, Faculty of Health, Iran University of Medical Sciences, Tehran, Iran; ^7^Department of Medical Urgent, Ministry of Health, Tehran, Iran; ^8^Research Center for Addiction & Risky Behavior (ReCARB), Psychiatric Department, Iran University of Medical Sciences, Tehran, Iran

## Abstract

This study is a sociological analysis of the three dimensions of social structure including institutional, relational, and embodied structures that have an impact on the individuals' deviant behaviors in the society. The authors used a mix method to analyze the qualitative and quantitative data of 402 high risk abandoned substance users in 2008 in Tehran, capital city of Iran. The leading reasons of substance use were categorized into four fundamental themes as follows: stress, deviant social networks, and low social capital and weak social support sources. In addition, the epidemiology model of regression analysis provides a brief explanation to assess the association between the demographical and etiological variables, and the drug users' deviant behaviors. In sum, substance use is discussed as a deviant behavior pattern which stems from a comorbidity of weak social structures.

## 1. Introduction

Housing and treating homeless addicts is one of the major sociomedical policies worldwide. Iran is located in western Asia, with over 77 million inhabitants and also more than two-thirds of the population is under the age of 30 with one-quarter being 15 years of age or younger. Iran also exhibits one of the steepest urban growth rates in the world and approximately over 70 percent of Iran's population lives in urban areas. Moreover, the indicators for health and education have improved dramatically during the recent years [1].

On the other hand, Iran is one of Afghanistan's neighbors and therefore has the most serious problems in Asia. The latest rapid situation assessment of substance use in 2011 estimates the number of dependent substance users in Iran at 1,200,000, corresponding to 2.2% of the adult population [2] and a rise in injection drug use has a worrying ascending trend [1].

A brief report of substance abuse policy in Iran shows that government authorities and special services for treatment and harm reduction and prevention are being implemented on a patient-based approach [3].

Despite a significant increase in antisubstance trafficking efforts and establishment of several treatment centers in recent decades, the number of substance users has had an ascending trend and the age of onset has decreased [4].

Social policymakers have discovered a structural understanding of individual and social factors related to various levels of social health, providing an insight that can be used as the basis of public health policies [5]. However, the application of evidence-based thinking in primary prevention is definitely hampered by the complexity of the causal chain. In addition, “the knowledge about the first link is uncertain because of social and psychological factors” [6]. In addition, to identify effective strategies, evidence-based prevention programs and strategies adaptation need to be better understood as well as factors associated with institutionalization of effective prevention programs [7]. Thus, the main aim of this study is to investigate the most important social factors affecting substance use and other deviant behaviors in this country; creating a structural discussion on the pathology of this phenomenon.

## 2. Materials and Methods

This survey was implemented as a prospective study on 402 high risk abandoned substance users admitted to Shafagh Rehabilitation Center: a clinical and psychological treatment center affiliated to the Ministry of Health, in collaboration with the Police Department and Iran's Drug Control Headquarters in the year 2008 in Tehran [7]. A standard questionnaire was designed by researchers and experts to estimate baseline characteristics, sociodemographic variables, drug users' experiences during rehabilitation treatment, imprisonment period, and substance use causes. Narcotic replacement therapy at Shafagh Center was based on methadone therapy for 6 months.

### 2.1. Data Collection

Upon entrance to the rehabilitation center, the aim of the interview was explained to subjects and, after obtaining consent to participation, the questionnaire was completed for each individual via a face-to-face interviews, which were in-depth, semistructured interviews conducted by 3 social workers and 1 clinical psychologist [7, 8]. Qualitative data that were collected through semistructured interviews including the subjects' self-report reasons regarding substance use were extracted via the following questions: “How did you get involved in substance addiction?” and “What was your main reason of substance use?”. Field notes were analyzed using a thematic analysis with inductive hand coding in order to derive themes by two researchers. This work was designed to construct the theories that are grounded in the data themselves. Indeed, the process of coding occurred without trying to fit the data into a preexisting model or frame. This process mainly consisted of reading transcripts, generating initial codes, comparing and contrasting themes, and building theoretical models and thereafter developing a mixed methodology by entering qualitative information into a computer at the first opportunity.

### 2.2. Data Rigor and Trustworthiness

The rigor and trustworthiness of the data were ensured through immersion in the subject, peer checking, and data source triangulation using experts from different fields for collection. With the aim of conducting a peer review, each interview was first coded by the first author and then reviewed by the second author who modified the manuscript if necessary. Moreover, the charts extracted by the first author were checked by the second author in the middle and late stages of the analysis and extracted; the themes, subthemes, and related statements are provided.

### 2.3. Statistical Analysis

Descriptive statistics such as number and percent for categorical and mean ± SD for continuous variables was used for descriptive tables. Also, in the analytic statistics, univariate logistic regression was used to assess the associations between drug use and demographical and etiological variables. The *P* value of less than 0.05 was considered statistically significant. All analyses were performed using SPSS software version 16.

### 2.4. Ethical Considerations

The data collected in the present study is a part of the data obtained for a research project approved by the Health Ministry of Iran. The study participants submitted their informed consent forms after the study objectives were described to them and after confidentiality and anonymity of their information were assured. Moreover, at interviews, all ethical principles and subjects' rights to withdraw from the study at any stage were observed.

### 2.5. Theoretical Framework

**Figure 1:**
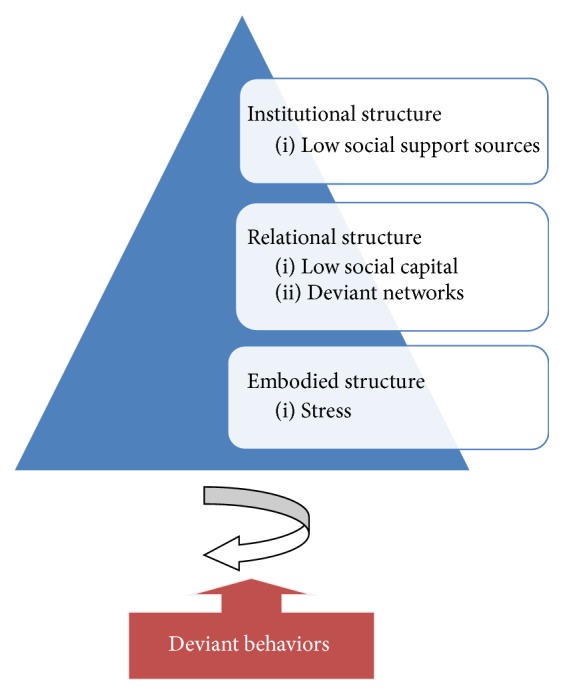
The study theoretical framework.

After contrasting, the study themes of the theoretical framework were borrowed from the Social Ecological Theory developed by Berkowitz and Perkins (1986) which explains the causes of substance abuse as being within the social environment and the social group in which individuals interact [9]. It is hypothesized that, to change a particular behavior, the social context that shapes it must be changed and therefore, to change the behavior, the social institutions that shape it must undergo change. Prevention efforts using this theory focus on changing the environment and mainly the socialization process rather than the person [9]. In addition, this study is referred to as the “Social Stress Model of Substance Abuse” and theorizes that the probability of engaging in drug use is assessed as a function of the stress level and to the extent of which it is triggered by stress moderators, social networks, social competencies, and resources of communities [10]. Moreover, in a sociological view, López and Scott's idea of the triple concept of social structure (2000) including institutional, relational, and embodied structures has provided a useful framework for understanding the impacts of combined social structure on deviant behaviors [11]. Drawing on the above theories, the fundamental variables of influencing deviant behaviors were defined in three structural levels and four categories in Figure 1 and the following.

*Etiologic Variables of Drug Use*

*Variables (Themes and Codes)*

*Deviant Social Networks*

- Having a drug user in peers group.
- Having a drug user in family.
- Having an imprisonment history.
- Joining a gang.

*Low Social Capital*

- Immigration.
- Being away from family.
- Divorce.
- Family disputes and tensions.
- Intragroup conflicts.
- Loss of close relatives and friends.

*Weak Social Sources*

- Joblessness.
- Severe life conditions.
- Economic hardship.
- Adverse living conditions.
- Unavailability of appropriate medical facilities.
- Inappropriate welfare facilities.
- Risky environment.
- Easy access to drugs.

*Stress*

- Feeling of loneliness.
- Feeling of hopelessness.
- Feeling of worthlessness.
- Feeling of anxiety.
- Feeling of fearfulness.

*Statements *

- “I had never lived with the fear that I had no friends, so I did almost anything to keep the two good friends I still had. “When I was 17, I started smoking cigarettes and alcohol because of my friends. At age 18, I started getting into and hanging out with gang members.” I started acting out like my peer drug addicts, stealing and sneaking out at night.”
- “Both of my parents are active addicts, and they abused me physically and verbally, it was my father who got me into drugs.”
- “My parents were getting a divorce when I was 14. We moved to new city, I was isolated and they did not care for me. I started using alcohol and drugs with my new friends after school.”
- “I had many problems with my parents. Little by little, the condition became worse. Next year I was expelled from school. My dad and I got into a fight. A friend said drug was a source of relaxation. It helps you fit in or make you cool.”
- “We had many problems in our life. I was kicked out of my work for a year. I was jobless and my pockets were empty. I needed something to be cool.”
- “I got sick and my family was poor. I didn't know how can treat my sharp pain. It was getting worse. I was motivated to use drugs.”
- “I was so lonely and anxious. It led me to use drugs to gain a pleasant feeling. I heard just try crack once and everything's gonna be okay; it will make everything go away.”
- “I struggled with depression and hopelessness for a long time. My friends used the substance; they were enjoying themselves and bonding.”
- “I rarely had a friend and I was so alone in school and I thought to be worthlessness and boring for others. The fear of loneliness led me to smoke cigarette in the park as a common action that shows I am like you, then use alcohol in a party and step by step I found that I am a drug user and in addiction.”

## 3. Results and Discussion

As can be seen in Table 1, among 402 reported drug users, 386 (96.5%) of them were men and 294 (73.4%) subjects were single or divorced. Moreover, the majority of drug users were in the age range between 20 and 39 years, with a mean age of 28.78 years and minimum and maximum of 13 and 62 years, respectively. Concerning the education level, majority of drug users, 339 (87.1%), had primary or secondary school education diplomas and also 15 (3.9%) subjects were illiterate. Early onset of drug use in most participants (57.6%) was ≤20 years with an average age of 21.21 and also a minimum and maximum age of 17 and 53 years, respectively (SD = 6.363).

In addition, 213 (57.1%) of participants had the minimum of a 5-year period of drug use. However, the majority of substance users had a history of addiction treatment and just 37% of them were treated by a physician. In addition, other risky behaviors including needle sharing, pre- or extramarital sex, and condomless sex were common among 21 (5.2%), 43 (10.7%), and 38 (19.5%) of drug users, respectively.

On the other hand, the majority of substance users did not report a history of severe physical and mental illness before the onset of drug use (87.1% and 82.6%, resp.).

The subjects also reported the following substance uses: cigarettes, 385 (95.8%); opium, 321 (79.9%); heroin, 259 (66.4%); crack, 227 (56.7%); cannabis, 174 (43.3%); alcohol, 164 (40.5%); and sedatives, 117 (29.1%).

In addition, the reported major reasons were categorized into four main themes including deviant social networks (26.2%), low social capital (16.5%), weak social sources (15.2%), and stress (37.1%).

According to the results of the univariate logistic regression test, in Table 2, there are significant associations among the variables of age (OR = 1.04; 95% CI: 1.01–1.07), divorce status (OR = 2.07; 95% CI: 1.23–3.49), and the history of imprisonment (OR = 2.12; 95% CI: 1.32–3.40) with presence of cannabis use. Moreover, diploma or academic educational level (OR = 0.26; 95% CI: 0.07–0.94) and history of imprisonment (OR = 0.35; 95% CI: 0.21–0.58) have significant protective association with presence of alcohol consumption. Also, there is a significant protective association between primary or secondary educational level (OR = 0.29; 95% CI: 0.14–0.60) and history of imprisonment (OR = 0.27; 95% CI: 0.17–0.44) and the presence of heroin consumption. Diploma or academic educational level is identified as a protective factor (OR = 0.29; 95% CI: 0.14–0.60) in comparison to lower education levels for presence of cocaine consumption. Also, there is a significant protective association between history of imprisonment (OR = 0.49; 95% CI: 0.28–0.84) and sedative drug consumption.

Moreover, in Table 3, the findings provided are regarding the psychosomatic indicators that have been discovered after six months of methadone therapy and an education program for social-emotional skills. These results have shown that this program was approximately successful in decreasing drug users' stress indicators [8].

### 3.1. Discussion

During recent years, there has been a considerable development in science-based prevention, providing new prevention models such as those based on risk and protective factors. In fact, the most important aspects of the models are their prognostic value and their influences on the success of treatment program [6, 12].

### 3.2. Comorbidity of High Stress, Low Social Capital, and Deviant Networks

According to the study qualitative findings derived from the drug users' self-reported statements, the most important reasons of substance use were prioritized as stress that could be associated with a comorbidity of other external stressors and the pressure of deviant social networks, low social capital, and weak social sources in the society. Other studies confirm an increasing adolescents' stress in Iran's contemporary society [13, 14]. Furthermore, pleasure seeking and release of tension are discussed as the most common reasons for substance use among Iranian high school students [15].

On the other hand, the drug users' psychotic symptoms assessment after a 6-month period of treatment and educational interventions confirmed a decline of some symptoms such as the feeling of tension, hopelessness, worthlessness, nervousness, and suicidal thoughts. These mental-social disorders may be interpreted as the weakness of emotional and social skills [6, 16]. Other studies show how high school dropouts are two to three times more likely to begin and maintain injecting drug use, compared to high school graduates [17, 18]. It seems that the disruption and dysfunction of leading social institutions such as the family and school may have led to a major deprivation in individuals' in both emotional and social skills and lead to fundamentally a weak embodied structure to cope with complex issues in the contemporary world.

Based on this study's findings, most participants are single and without a high educational level (87.1% below high school diploma), indicating a high rate of school dropouts and the decline of intergroup social capital as a risk factor in the drug users' socialization, particularly, because 57.6% of them have started drug abuse at the age below 20 years and, in sum, 16.5% of them are marked by low integration into a family and 57.3% of them suffered from “a feeling of loneliness.” In addition, the other studies show a low range of social capital within the drug users [19]. Indeed, bonds with friends and family are the strongest differentiating factors between substance users and nonusers [20]. Confirming the World Health Organization (2001) research findings on risk and protective factors from more than 50 countries, the most common risk factor for adolescent substance use in Asia is the conflict within the family and friends who use substances. It is also concluded that a positive relationship with parents, parents who provide structure and boundaries, and a positive school environment are the most leading protective factors [21, 22].

Social and emotional skills learning as a unifying theory with an increasing body of research demonstrates evidence-based interventions which are associated with healthy behaviors [23–26].

### 3.3. Weakness of Social Sources

On the other hand, the other extracted indictors of this study such as joblessness and severe life conditions, economic hardship, adverse living conditions, unavailability of appropriate medical facilities, and inappropriate welfare facilities can be demonstrated to a large extent as the weakness of social support sources.

According to the findings, there is an addiction period of more than 5 years among 57% of the drug users and most of them have given up drugs several times, but approximately only 30% of them had been under treatment by a physician. These findings confirm previous studies regarding the high relapse rate and more common behavioral changes as an active and multidimensional process in which the clients experience a psychological status spectrum from recovery to the relapse complex: a process influenced by the treatment process and individual factors associated with the patients [27, 28].

It can be hypothesized that there exists a low coverage level of therapeutic services for substance in Iran, since some participants stated running out of medicines or not being able to repurchase them as their main reasons for the noncommitment to the principles of the treatment program resulting in a relapse to addiction. There is a necessity for promoting the level of service utilization and continuation of medical and nonmedical treatment services in primary health care.

In addition, low levels of social support system, such as joblessness, financial difficulties, and lack of social welfare in a society, are the significant indicators that can impact and shift social policies toward the improvement of wellbeing and social health [9, 10, 12]. The second explanation for the association between mental disorders and poor social circumstances is that individuals in socially disadvantaged situations are exposed to more psychosocial stressors (adverse life events) than those in more advantageous environments. These stressors act as triggers for the onset of symptoms and the loss of the individual psychological abilities necessary for social functions [29, 30].

According to the results of the regression analysis, being older and divorced and a history of imprisonment have an impact only on cannabis users as a risk factor and played a protective role on the other drug users.

Moreover, diploma or academic educational levels and history of imprisonment were protective factors associated with alcohol consumption. Also, primary or secondary educational levels and a history of imprisonment were protective factors for heroin users. Diploma or academic educational levels were identified as protective factors for cocaine users. A history of imprisonment was a protective factor for sedative drug consumption. Regarding the effectiveness of previous imprisonment on drug users, it is confirmed by findings of various studies [31]. A relapse to drugs and alcohol abuse occurred in a context of poor social support, medical comorbidity, and inadequate economic resources [32]. However, a strong systemic provision on the access to drugs and the prison-based residential drug and alcohol treatment programs has been suggested in prisons. The above findings confirm the combination of this study's qualitative findings, especially for drug users who have a higher education level.

However, substance use and addiction treatment is not widely available in prisons and studies support that most people with substance abuse issues who are released from prison relapse once back in the community. The prevention interventions after prison for drug users may include structured treatment with gradual transition to the community, improved protective factors, and reductions of environmental risk factors [32].

## 4. Conclusion

According to the study findings, there is a comorbidity of various risk factors including the weakness of social capital, deviant social networks, and a low stock of social sources that overall have an impact on the risk factors of drug use. However, in the regression analysis, the results of some variables have not clarified the associations strongly because of the small sample size of this study that again alerted smaller subgroups through distributing them among the users of drug types. In sum, based on these findings, it can be concluded that a major part of drug users commonly use substances as a way to deal with difficulties in stressful situations. And, from a social point of view, substance use is acknowledged as a deviance behavior which stems from the functional weakness of institutional, relational, and embodied structures.

Thus, this study illuminated the need for a focus on the contributions of policymakers to developing strategies in improving social sources, social capital within the family unit and prosocial networks, and enhancing health services.

## Figures and Tables

**Table 1:** Baseline characteristic of participants.

Variables	Frequency *n* (%)
*Sex* (399)	
Male	386 (96.5)
Female	14 (3.5)
*Age (years)* (382)	
20–29	148 (38.7)
30–39	164 (42.9)
≥40	70 (18.3)
*Marital status* (387)	
Single	189 (46.3)
Divorce	109 (27.1)
Married	92 (22.9)
*Educational level* (389)	
Illiterate	35 (9.0)
Primary/secondary school	239 (61.4)
High school	100 (25.7)
University	15 (3.9)
*Occupation *	
Yes	328 (85.2)
No	57 (14.8)
*Age of onset* (382)	
≤20	220 (57.6)
21–30	129 (33.8)
≥31	33 (8.6)
*Addiction duration* (years) (370)	
≤5	160 (42.9)
≥6	213 (57.1)
*Addiction treatment history* (383)	
No	67 (17.5)
Yes	316 (82.5)
*Medical treatment for addiction *	
Yes	104 (30.7)
No	235 (69.3)
*Imprisonment history* (381)	
Yes	277 (72.7)
No	104 (27.3)
*Needle sharing*	
Yes	21 (5.2)
No	381 (94.8)
*Pre- or extramarital sex*	
Yes	43 (10.7)
No	359 (89.3)
*Condomless sex*	
Yes	38 (19.5)
No	364 (90.5)

**Table 2:** Univariate logistic regression for assessing associations between drug use and related factors.

Variables	Cannabis OR^*∗*^ (95% CI)	Alcohol OR (95% CI)	Opium OR (95% CI)	Heroin OR (95% CI)	Cocaine OR (95% CI)	Sedative OR (95% CI)
*Age*	**1.04 (1.01–1.07)**	1.01 (0.99–1.01)	0.97 (0.94–1.01)	0.98 (0.96–1.01)	1.08 (0.92–1.05)	0.98 (0.95–1.01)
Sex						
Female	Reference	Reference	Reference	Reference	Reference	Reference
Male	1.61 (0.14–17.98)	1.45 (0.13–16.10)	9.55 (0.85–106.87)	1.01 (0.09–11.26)	1.71 (0.15–16.54)	1.16 (0.10–13.00)
*Marital status*						
Single	Reference	Reference	Reference	Reference	Reference	Reference
Divorced	**2.07 (1.23–3.49)**	1.42 (0.85–2.39)	0.81 (0.38–1.74)	1.64 (0.98–2.75)	6.35 (0.39–9.45)	0.61 (0.11–3.38)
Marriage	1.34 (0.83–2.15**)**	0.89 (0.55–1.44)	1.10 (0.57–2.12)	0.81 (0.48–1.38)	1.03 (0.29–3.60)	0.33 (0.07–1.49)
*Educational level*						
Illiterate	Reference	Reference	Reference	Reference	Reference	Reference
Primary/secondary school	0.54 (0.25–1.14)	0.53 (0.25–1.15)	1.01 (0.39–2.59)	**0.29 (0.14–0.60)**	**0.29 (0.14–0.60)**	0.54 (0.22–1.27)
Diploma/academic	0.40 (0.11–1.38)	**0.26 (0.07–0.94)**	1.20 (0.25–5.60)	0.33 (0.09–1.18**)**	0.33 (0.09–1.18)	1.62 (0.29–8.92)
*History of imprisonment*						
No	Reference	Reference	Reference	Reference	Reference	Reference
Yes	**2.12 (1.32–3.40)**	**0.35 (0.21–0.58)**	0.87 (0.49–1.56)	**0.27 (0.17–0.44)**	1.00 (0.26–3.84)	**0.49 (0.28–0.84)**
*Deviant network*						
No	Reference	Reference	Reference	Reference	Reference	Reference
Yes	0.88 (0.49–1.50)	0.55 (0.49–1.57)	1.10 (0.52–2.30)	1.13 (0.61–2.10)	0.48 (0.10–2.21)	1.06 (0.54–1.85)
*Low social capital*						
No	Reference	Reference	Reference	Reference	Reference	Reference
Yes	0.85 (0.44–1.60)	1.96 (0.99–3.86)	0.38 (0.13–1.13)	0.97 (0.48–1.96)	1.45 (0.17–12.42)	1.31 (0.64–2.65)
*Low social sources*						
No	Reference	Reference	Reference	Reference	Reference	Reference
Yes	1.53 (0.83–2.83)	1.09 (0.60–1.99)	0.83 (0.37–1.87)	1.59 (0.85–2.96)	1.93 (0.22–16.38)	0.88 (0.47–1.64)
*Stress*						
No	Reference	Reference	Reference	Reference	Reference	Reference
Yes	0.87 (0.49–1.54)	0.99 (0.56–1.75)	1.69 (0.84–3.40)	0.60 (0.31–1.15)	1.06 (0.19–5.31)	0.86 (0.47–1.55)

^*∗*^Odds ratio.

**Table 3:** The drug users' psychotic symptoms after methadone therapy and learning social-emotional skills for six months.

Variables (*n*)	Never *n* (%)	Seldom *n* (%)	Sometimes *n* (%)	Often *n* (%)	Always *n* (%)
Feeling of hopelessness (390)	283 (72.6)	14 (3.6)	59 (15.1)	19 (4.9)	15 (3.8)
Feeling of loneliness (390)	139 (35.5)	27 (6.9)	146 (37.3)	55 (14.1)	23 (5.9)
Feeling of worthlessness (392)	201 (51.3)	25 (6.4)	118 (30.1)	30 (7.7)	18 (4.6)
Feeling of nervousness (389)	175 (45.0)	55 (14.1)	88 (22.6)	54 (13.9)	17 (4.4)
Feeling of fearfulness (390)	155 (39.7)	30 (7.7)	164 (42.1)	28 (7.2)	13 (3.3)
Having suicidal thoughts (390)	295 (75.6)	38 (9.7)	33 (8.5)	18 (4.6)	6 (1.5)
